# Analytical Method to Estimate the Complex Permittivity of Oil Samples

**DOI:** 10.3390/s18040984

**Published:** 2018-03-26

**Authors:** Lijuan Su, Javier Mata-Contreras, Paris Vélez, Armando Fernández-Prieto, Ferran Martín

**Affiliations:** 1 CIMITEC, Departament d’Enginyeria Electrònica, Universitat Autònoma de Barcelona, Bellaterra, 08193 Cerdanyola del Valldès, Spain; sulijuan1020@163.com (L.S.); FranciscoJavier.Mata@uab.cat (J.M.-C.); ferran.martin@uab.cat (F.M.); 2 Departamento de Electrónica y Electromagnetismo, Universidad de Sevilla, Av. Reina Mercedes s/n, 41012 Sevilla, Spain; armandof@us.es

**Keywords:** complex dielectric constant, complex permittivity measurements, microwave sensors, complementary split ring resonator (CSRR), microstrip technology

## Abstract

In this paper, an analytical method to estimate the complex dielectric constant of liquids is presented. The method is based on the measurement of the transmission coefficient in an embedded microstrip line loaded with a complementary split ring resonator (CSRR), which is etched in the ground plane. From this response, the dielectric constant and loss tangent of the liquid under test (LUT) can be extracted, provided that the CSRR is surrounded by such LUT, and the liquid level extends beyond the region where the electromagnetic fields generated by the CSRR are present. For that purpose, a liquid container acting as a pool is added to the structure. The main advantage of this method, which is validated from the measurement of the complex dielectric constant of olive and castor oil, is that reference samples for calibration are not required.

## 1. Introduction

This paper deals with microwave sensors based on transmission lines loaded with resonant elements. Within this topic, split ring resonators (SRRs) [1] and their complementary counterparts, complementary split ring resonators (CSRRs) [2], have been extensively used as sensing elements due to the high sensitivity of their electromagnetic properties to the surrounding medium [3,4,5,6,7,8,9,10,11,12,13,14,15,16,17,18,19,20,21,22,23,24,25,26,27,28,29,30,31,32,33,34]. Three main sensing strategies in SRR or CSRR-loaded lines have been considered: (i) variation in the notch frequency and depth caused by resonator loading (including dielectric loading) [3,4,5,6,7,8,9,10,11,12,13,14,15]; (ii) frequency splitting in transmission lines loaded with pairs of resonant elements, which is caused by asymmetric dielectric loading [16,17,18,19,20,21,22,23]; and (iii) coupling modulation sensors, where the notch depth is controlled by symmetry disruption [24,25,26,27,28,29,30,31,32,33,34].

Coupling modulation sensors are based on symmetry properties. Such sensors are implemented by symmetrically loading a host line with a symmetric resonator. The line and resonant element must be selected such that in the unperturbed state (perfect symmetry), line-to-resonator coupling is prevented. This is achieved if the symmetry planes of the line and resonator are of a different electromagnetic sort, i.e., one of them is a magnetic wall, and the other one is an electric wall. For instance, if the host line is implemented in coplanar waveguide (CPW) technology, where the line axis (symmetry plane) is a magnetic wall for the fundamental CPW mode, a convenient resonator is the SRR, since it exhibits an electric wall (symmetry plane) at the fundamental resonance frequency [24]. Coupling modulation sensors have been mainly applied to the measurement of spatial variables and velocities by etching the resonant element in a movable substrate, with regard to the one of the host line [27,28,29,30,31,32,33,34].

In frequency-splitting sensors, a transmission line (or a more complex, but symmetric, structure) is symmetrically loaded with a pair of (not necessarily symmetric) resonators. In the unperturbed state (i.e., by preserving the symmetry), a single notch in the transmission coefficient appears. However, if symmetry is disrupted, e.g., by means of an asymmetric dielectric loading, then two notches arise, and the distance between the notches is related to the level of asymmetry. This type of sensor has been used for the measurement of the complex dielectric constant of liquids, particularly mixtures of Deionized (DI) water and ethanol [23]. Reference liquids with well-known dielectric constant are needed for calibration purposes, and the dielectric properties of the liquid under test (LUT) are obtained from the frequency distance and the difference in notch depth between the two resonances (notches) that is generated when the reference liquid and the LUT are different (producing an asymmetry).

The most simple and extended microwave sensors implemented by means of resonator-loaded lines are those based on the resonance frequency and notch depth variation. In this case, a single resonator electromagnetically coupled to the line is needed, and no further requirements relative to symmetry apply. Any kind of perturbation in the surrounding medium of the resonant element that is able to alter the resonance frequency and/or notch depth is susceptible to be sensed. As compared to the previous sensors (frequency-splitting and coupling modulation sensors), these sensors are less immune to cross-sensitivities (e.g., those caused by ambient factors such as moisture or temperature), and hence are less robust against the effects of environmental changes. Under some circumstances, this is not an issue. Nevertheless, these sensors in general need calibration, using reference samples with well-known dielectric properties, provided that the application is focused on the measurement of the complex dielectric constant of materials.

In this paper, we present a sensor based on the variation of the notch depth and frequency of a resonator-loaded line, which is useful for the measurement of the complex dielectric constant of materials. Although this strategy for sensing (based on notch depth and frequency variation) is not new, the sensing approach for determining the complex dielectric constant is unconventional, since it is based on an analytical method, and calibration is not needed. However, certain requirements related to the characteristics of the line and material under characterization (to be discussed) must be satisfied. The sensing strategy is based on the analysis carried out by Su et al. in [13], and the method was first applied by Su et al. in [14] for the estimation of the complex dielectric constant of liquids (DI water). In this paper, a novel structure that is specifically suited to determine the dielectric constant and loss tangent of oil samples at the S-band is reported. Additionally, the analytical sensing method is described in detail, and its limitations are discussed.

## 2. Description of the Proposed Sensor, Working Principle, and Sensing Method

**Figure 1:**
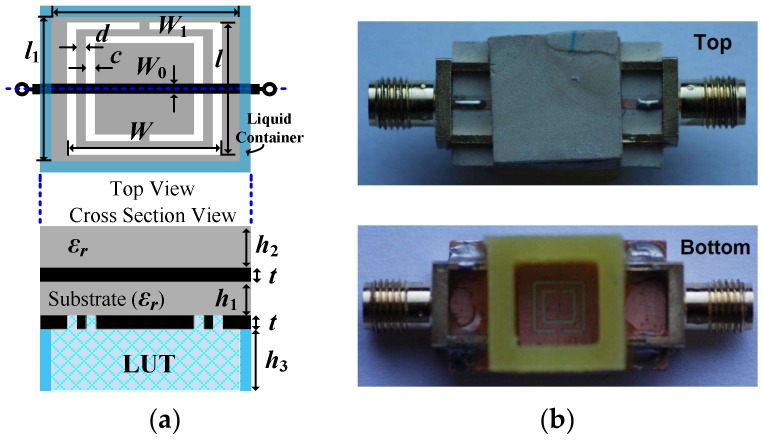
Top and cross-section views (**a**) and photograph (**b**) of the complementary split ring resonator (CSRR)-loaded embedded microstrip line. Dimensions are: *W* = *l* = 5.92 mm, *c* = *d* = 0.5 mm, *W*_0_ = 1.15 mm, *W*_1_ = *l*_1_ = 9.92 mm, *h*_1_ = 1.27 mm, *h*_2_ = 3.81 mm, *h*_3_ = 7.2 mm, and *t* = 35 μm. The substrate is *Rogers RO3010* with dielectric constant *ε_r_* = 10.2, and the container is FR4.

The proposed sensing structure is depicted in Figure 1. It consists of an embedded microstrip line loaded with a square-shaped complementary split ring resonator (CSRR), which acts as the sensing element. The main advantage of using a CSRR is that this resonator exhibits a distributed capacitance along the whole perimeter, and for this reason, its capacitance is quite sensitive to the presence of a LUT on top of it. In other words, the notch frequency is quite sensitive to the permittivity of the LUT. Moreover, a liquid container is added to the ground plane surrounding the CSRR. By introducing the LUT in such a container, it is possible to achieve certain liquid depth, which is necessary in the proposed sensing method (particularly, the depth of the container is achieved if it is completely full with the LUT). Indeed, the validity of the method is based on the homogeneity of the dielectric surrounding the metallic strip of the line, and also on the homogeneity of the dielectric material (liquid in our case) surrounding the CSRR. Obviously, such homogeneity is necessary only in the regions where the electromagnetic fields generated by the line and CSRR are present. For this reason, the considered line is an (unusual) embedded microstrip line. The thickness of the dielectric layer on top of the line, *h*_2_, which is indicated in the caption of Figure 1, is enough to ensure that the field lines do not reach the air/dielectric interface. The height of the liquid pool, *h*_3_, which is also indicated in the caption of Figure 1, has been chosen to guarantee the uniformity of the material (LUT) on top of the CSRR (a minimum LUT depth is necessary so as to ensure that the electric field lines generated in the slots of the CSRR do not cross the air/LUT interface).

The working principle of the proposed sensor is based on the variation experienced by the notch position and depth in the transmission coefficient when the LUT is introduced in the container. The presence of the liquid modifies (increases) the capacitance of the CSRR (through the effects of its dielectric constant, *ε_LUT_*), and consequently, the resonance frequency of this element shifts down. On the other hand, the notch depth is intimately related to losses, and such losses are influenced by the loss tangent (tan*δ_LUT_*) of the LUT. Therefore, the determination of such a loss parameter (tan*δ_LUT_*) is possible. Obviously, by obtaining the variation of the notch frequency and depth of liquid samples with well-known complex permittivity, calibration curves can be generated, and the measurement of the dielectric constant and loss tangent of the LUT can be achieved [23]. In this paper, we propose an unconventional method in which the measurement of such material parameters does not require any calibration (as long as the measurement of the dielectric constant and loss tangent of the LUT is done from an analytical method). However, the complex permittivity of the substrate material must be accurately known, as usual in low-loss commercial microwave substrates. Note that the use of low-loss substrates (i.e., with a small loss tangent) is necessary, especially if the materials under test exhibit moderate or low losses. Otherwise, substrate losses may degrade the sensitivity of the method for the measurement of material losses (LUT in our case).

**Figure 2:**
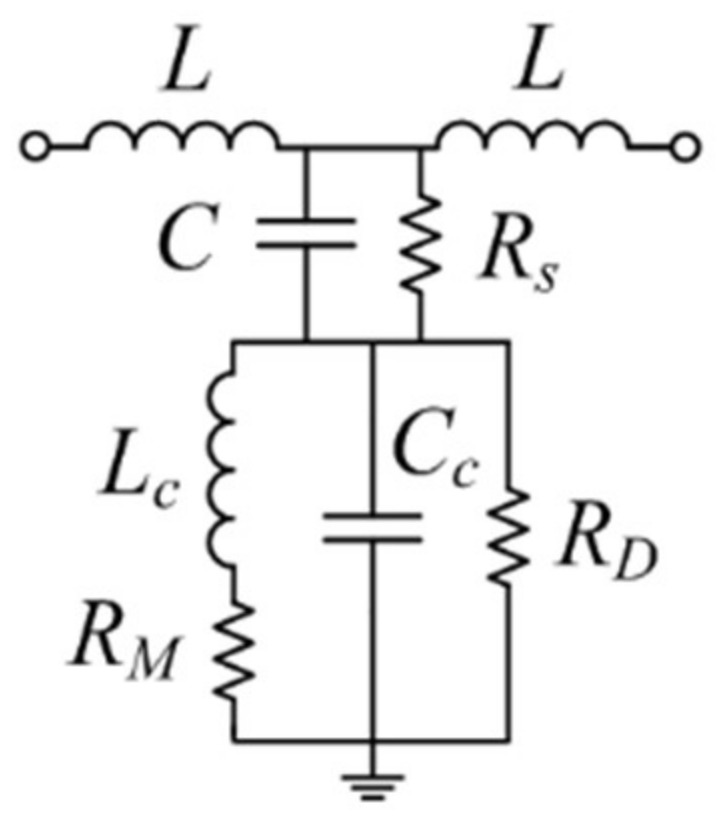
Lumped element equivalent circuit of the CSRR-loaded embedded microstrip line, without liquid in the container.

The sensing method, which will be discussed next, is based on the lumped element equivalent circuit model of the structure of Figure 1, which is depicted in Figure 2. Concerning the reactive elements of the model, *L_c_* and *C_c_* are the inductance and capacitance, respectively, of the CSRR, *L* models the inductance of the conductive strip (line), and *C* accounts for the capacitance between such strip and the inner region of the CSRR. Losses include substrate losses, through *R_s_*, plus CSRR losses, modeled by *R_M_* (ohmic losses) and *R_D_* (dielectric losses of the substrate). Radiation losses are excluded, since electromagnetic simulations of the structure by excluding dielectric and ohmic losses (not shown) indicate that unitarity is preserved in the region of interest. The sensing method is based on the following equations [11]:

(1) $$R_{eq}=\frac{1}{R_{S}C^{2}\omega^{2}}+\frac{R_{M}R_{D}^{2}+R_{D}L_{c}^{2}\omega^{2}}{R_{D}^{2}{\left(1-L_{c}C_{c}\omega^{2}\right)}^{2}+{\left(L_{c}\omega +R_{M}R_{D}C_{c}\omega \right)}^{2}}$$

(2) $$\chi_{eq}=\frac{-1}{C\omega }+\frac{R_{D}^{2}L_{c}\omega (1-L_{c}C_{c}\omega^{2})-R_{M}R_{D}(L_{c}\omega +R_{M}R_{D}C_{c}\omega )}{R_{D}^{2}{\left(1-L_{c}C_{c}\omega^{2}\right)}^{2}+{\left(L_{c}\omega +R_{M}R_{D}C_{c}\omega \right)}^{2}}$$

where *ω* is the angular frequency. The previous equations provide the real (1) and imaginary (2) part of the impedance of the shunt branch. Another necessary equation in this analytical method is the expression providing the magnitude of the transmission coefficient at the frequency, *ω*_0_, where *χ_eq_* = 0, that is, [11]:

(3) $${\left|S_{21}\right|}_{\omega_{0}}=\frac{2Z_{0}R_{eq}}{\sqrt{{\left(2Z_{0}R_{eq}+Z_{0}^{2}-L^{2}\omega^{2}\right)}^{2}+{\left[2L\omega (Z_{0}+R_{eq})\right]}^{2}}}$$

with *Z*_0_ being the reference impedance of the ports.

**Figure 3:**
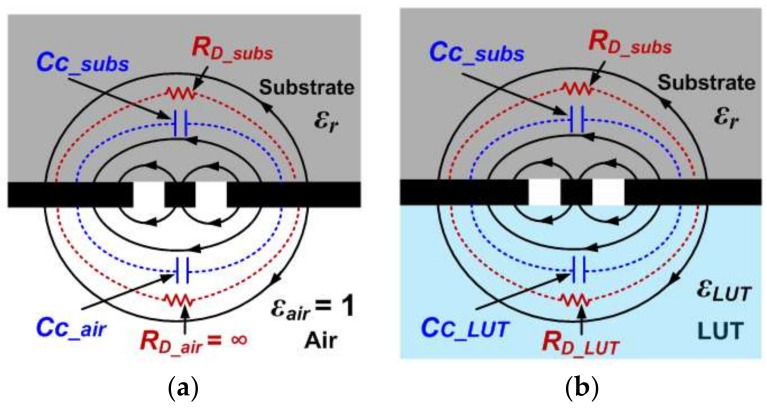
Cross-sectional view of the slot region of the CSRR, with electric field lines and contributions to the total CSRR capacitance and dielectric resistance. (**a**) Empty container; (**b**) full container.

Let us consider the structure of Figure 1 without LUT in the container (unloaded CSRR). From the measured frequency response, the reactive parameters (*L_c_*, *C_c_*, *L*, and *C*) can be inferred using the parameter extraction method reported by Bonache et al. in [35]. On the other hand, due to the uniformity of the dielectric material surrounding the strip line (with well-known loss parameter, tan*δ*), *R_S_* and *R_D_* can be obtained by means of [11]:

(4) $$\tan\delta =\frac{1}{Q_{s}}=\frac{1}{R_{S}C\omega }$$

(5) $$\tan\delta =\frac{1+\varepsilon_{r}}{R_{D}C_{c}\varepsilon_{r}\omega }$$

where *Q_s_* is the substrate quality factor, and *ε_r_* is the dielectric constant of the substrate. Equation (4) is strictly valid in an embedded microstrip line, assuming that slots are not present in the ground plane. In our case, this condition is not satisfied due to the presence of the CSRR. However, as long as the side dimension of the CSRR, *l*, is high compared to *c* and *d*, Expression (4) provides a good approximation of the loss factor of the substrate. Expression (5), on the other hand, is derived as follows. The capacitance of the unloaded CSRR (i.e., without LUT) is given by the contribution of the substrate, *C_c_subs_*, plus the contribution of the air region, *C_c_air_*, (see Figure 3a) i.e.:

(6) $$C_{c}=C_{c\_subs}+C_{c\_air}$$

Since the capacitance of the air region is related to the capacitance of the substrate by *C_c_subs_* = *ε_r_*·*C_c_air_*, it follows that:

(7) $$\varepsilon_{r}C_{c}=C_{c\_subs}(1+\varepsilon_{r})$$

The loss tangent of the substrate can be also expressed as:

(8) $$\tan\delta =\frac{1}{R_{D}C_{c\_subs}\omega }$$

where it is assumed that the field lines do not cross the air/substrate interface of the CSRR slots, and the metal thickness is neglected. The actual metal thickness is *t* = 35 μm, as indicated in the caption of Figure 1, but this thickness is small enough, and hence, the above assumption is justified. Note also that *R_D_* = *R_D_subs_* (provided air can be considered as a perfect isolator, with *R_D_air_* = ∞), and for this reason, *R_D_subs_* in the denominator of Equation (8) can be replaced by *R_D_*, as indicated. By isolating *C_c_subs_* in Equation (7) and introducing the resulting expression in Equation (8), Equation (5) is finally obtained. Thus, from equations (4) and (5), the dielectric loss parameters *Rs* and *R_D_* can be obtained. Finally, the ohmic resistance *R_M_* is obtained from the measurement of the transmission coefficient at *ω*_0,_ which is also given by Equation (3). From Equation (3), the real part of the shunt impedance, *R_eq_*, can be obtained, and from it, *R_M_* can be inferred using Equation (1).

Once *R_M_* is known, by loading the container with the LUT, all of the model parameters remain invariable except *C_c_* and *R_D_*, which are influenced by the properties of LUT. Let us consider that *C_c_* is the CSRR capacitance with the container empty (as defined before), and *C_c_*′ is the CSRR capacitance with the presence of the LUT. The notch (angular) frequencies in both cases are given by:

(9) $$\omega_{0}=\frac{1}{\sqrt{L_{c}(C+C_{c})}}$$

(10) $$\omega_{0}\prime =\frac{1}{\sqrt{L_{c}(C+C_{c}\prime )}}$$

The capacitance *C_c_*′ is given by:

(11) $$C_{c}\prime =C_{c\_subs}+C_{c\_LUT}=C_{c\_subs}+\frac{\varepsilon_{LUT}}{\varepsilon_{r}}C_{c\_subs}$$

where *ε_LUT_* is the dielectric constant of the LUT. With equations (7) and (11), *C_c_*′ can be expressed in terms of *C_c_* as follows:

(12) $$C_{c}\prime =C_{c}\left(\frac{\varepsilon_{r}+\varepsilon_{LUT}}{\varepsilon_{r}+1}\right)$$

By introducing Equation (12) in (10), the dielectric constant of the LUT can be isolated, resulting in the following expression:

(13) $$\varepsilon_{LUT}=1+\frac{\left(\omega \prime_{0}^{-2}-\omega_{0}^{-2}\right)}{L_{c}C_{c}}(1+\varepsilon_{r})$$

Therefore, from the measurement of the notch frequencies for the empty and full container, the dielectric constant of the LUT can be inferred using Equation (13):

The dielectric loss of the CSRR with the presence of the LUT, *R_D_*′, can be obtained from Equations (3) and (1), with *R_D_* replaced with *R_D_*′ in Equation (1). Since *R_D_* is known, the loss associated to the LUT, *R_D_LUT_*, can be inferred, and from it, we can obtain the loss tangent of the LUT. Namely:

(14) $$R_{D}\prime =\frac{R_{D}R_{D\_LUT}}{R_{D}+R_{D\_LUT}}$$

since *R_D_* = *R_D_subs_*, as indicated before. From Equation (14), *R_D_LUT_* can be isolated, and once *R_D_LUT_* is known, the loss tangent of the LUT can be inferred according to:

(15) $$\tan\delta_{LUT}=\frac{1}{R_{D\_LUT}C_{C\_LUT}\omega }$$

with *C_c_L__UT_* given by:

(16) $$C_{C\_LUT}=C_{c}\prime \frac{\varepsilon_{LUT}}{\varepsilon_{r}+\varepsilon_{LUT}}$$

## 3. Results

**Figure 4:**
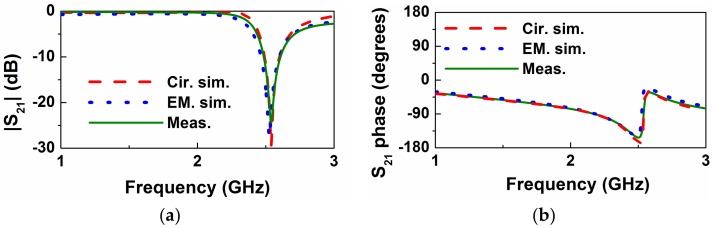
Transmission coefficient of the empty sensing structure of Figure 1. (**a**) Magnitude; (**b**) phase. Phase has been obtained after a reference plane shift.

The frequency response of the empty sensing structure of Figure 1 is depicted in Figure 4. This includes the measured response (inferred from the *Keysight* *N5221A* vector network analyzer), the electromagnetically simulated response (inferred from *CST Microwave Studio suite 2010*), and the circuit response (inferred from the element values of the circuit model, as shown in Table 1). The considered substrate, whose dimensions are indicated in the caption of Figure 1, has a dielectric constant of *ε_r_* = 10.2, and a loss tangent of tan*δ* = 0.0023. The measured response, with a notch frequency at *f*_0_ = *ω*_0_/2π = 2.54 GHz and a notch depth of −24.4 dB, is in very good agreement with the circuit simulation, pointing out the validity of the model (note that parameter extraction has been carried out from the experimental data).

**Figure 5:**
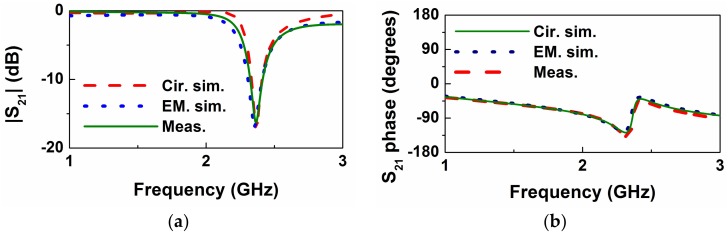
Transmission coefficient of the sensing structure with olive oil in the container. (**a**) Magnitude; (**b**) phase. Phase has been obtained after the reference plane shift.

By adding olive oil in the liquid container, the circuit response changes to the one indicated in Figure 5, where the notch frequency moves to *f*_0_′ = *ω*_0_′/2π = 2.37 GHz, and the measured notch level is found to be −16.26 dB. From these values, using Equation (13), the dielectric constant of the substrate is found to be *ε_LUT_* = 2.93. From equations (1) and (3), the dielectric loss of the CSRR with the presence of the LUT is found to be *R_D_*′= 530.84 Ω, and, using Equation (14), the resulting loss associated to the oil is *R_D_L__UT_* = 619.5 Ω. From Equation (15), using Equation (16), the loss tangent is found to be tan*δ_LUT_* = 0.103. The dielectric constant and loss tangent of olive oil obtained by the proposed method are in good agreement with independent results, which were measured with the dielectric probe kit *Keysight 85070E* at the resonance frequency of the loaded CSRR [15], i.e., *ε_r_* = 2.89, and tan*δ* = 0.116.

Let us briefly discuss the sources of errors in the previous results of *ε_LUT_* and tan*δ_LUT_*. On the one hand, the implementation of the embedded microstrip line in our in-house fabrication system has been done by attaching two commercially available substrates. This may somehow alter the uniformity of the substrate, as required. On the other hand, for the validity of Equation (4), the side dimension of the CSRR must be large compared to the width of the microstrip line and the thickness of the dielectric layer between the strip line and the ground plane. This condition is satisfied in our case to a good approximation, but some source of error in the determination of *R_S_* is expected. Nevertheless, these error sources are difficult to estimate.

Another error source comes from the tolerance in the value of the dielectric constant of the considered substrate, i.e., *ε_r_* = 10.2 ± 0.3 (or Δ*ε_r_* = ±0.3), according to the data sheet (the error in the loss tangent is not given, and therefore we will assume that this value, 0.0023, is accurate enough so as to neglect its uncertainty). The effects of Δ*ε_r_* on *ε_LUT_* can be inferred from Equation (13), where it can be seen that the corresponding error is simply:

(17) $$\Delta \varepsilon_{LUT}=\frac{\left(\omega \prime_{0}^{-2}-\omega_{0}^{-2}\right)}{L_{c}C_{c}}\Delta \varepsilon_{r}$$

and the evaluation of Equation (17) gives Δ*ε_LUT_* = ±0.05. Note also that the error in the dielectric constant of the substrate material influences *R_D_* (see Equation (5)). Replacing *ε_r_* with *ε_r_* ± Δ*ε_r_* in Equation (5), and assuming that Δ*ε_r_ << ε_r_*, the error in *R_D_* can be approximated as:

(18) $$\Delta R_{D}=\frac{\Delta \varepsilon_{r}}{\tan\delta \;C_{c}\varepsilon_{r}^{2}\omega }$$

giving Δ*R_D_* = ±9.74 Ω. Through conveniently propagating this error in Equation (1), one obtains *R_M_* = 0.0147 ± 0.00003 Ω, and finally, the error in the loss tangent is found to be Δtan*δ_LUT_* = ±0.0065.

**Figure 6:**
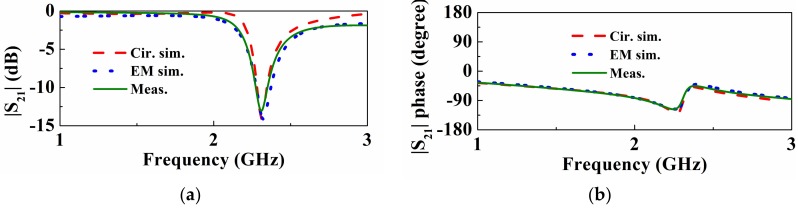
Transmission coefficient of the sensing structure with castor oil in the container. (**a**) Magnitude; (**b**) phase. Phase has been obtained after the reference plane shift.

We have also considered the estimation of the complex permittivity of castor oil. The corresponding response is depicted in Figure 6, where it can be appreciated that the notch frequency shifts down to *f*_0_′ = *ω*_0_′/2π = 2.31 GHz, and the measured notch level is found to be −13.05 dB. With these values, the analytical expressions of the method, and the previous error analysis, the resulting values of the dielectric constant and loss tangent are found to be *ε_LUT_* = 3.64 ± 0.07, and tan*δ_LUT_* = 0.139 ± 0.0060. The values measured with the dielectric probe kit *85070E* [15] are in this case *ε_r_* = 3.32, and tan*δ* = 0.105. Thus, the prediction of the method is not so good in this case, but it provides a reasonable estimation on account of the error sources mentioned (note that we have only taken into account the error generated by the tolerance in the dielectric constant of the substrate material, but not the other effects, which are difficult to evaluate in practice).

## 4. Discussion

Since the proposed method is based on the variation of the notch (resonance) frequency and depth, sensor sensitivities (which are defined as the variation of the notch frequency in relation to the dielectric constant of the LUT, *ε_LUT_*, and as the variation of the notch depth in relation to the loss tangent of the LUT, tan*δ_LUT_*) are intimately related to the dielectric constant, *ε_r_*, and the loss tangent, tan*δ*, of the substrate. This is very clear in light of Equation (13), where it can be appreciated that, for a certain value of *ε_LUT_*, a high value *ε_r_* with regard to *ε_LUT_* should provide a small difference between the resonance frequencies *ω*_0_ and *ω*_0_′. The dielectric constant of liquids is very diverse, from relatively small values in oil samples, to high values in DI water, for instance. Therefore, we have chosen a substrate with a moderate (with regard to the span values of liquid samples) dielectric constant of *ε_r_* = 10.2. Certainly, this is not the optimum solution for the measurement of oils. Nevertheless, the prediction of the dielectric constant in the considered oils is reasonable. Concerning the sensitivity in notch depth with loss tangent, from Equation (14), it can be seen that if substrate losses are high (i.e., small value of *R_D_*), then *R_D_*′, which is intimately related to the notch depth, will be necessarily small, and potential significant variations in *R_LUT_* (associated to tan*δ_LUT_*, see Equation (15)) will give small variation in *R_D_*′, and hence in the notch depth. In the considered substrate, the loss tangent is very small (tan*δ* = 0.0023), giving *R_D_* = 3709 Ω (see Table 1). This loss tangent is typically two orders of magnitude smaller than those of the oil samples, and therefore, the considered substrate is appropriate for the measurement of the loss factor in such liquids.

Concerning the sensitivity of frequency with the (dimensionless) dielectric constant, this value is given in several works that are devoted to the dielectric characterization of materials; so, that comparison is feasible. The corresponding (average) values, *S_av_*, are given in Table 2, where it can be appreciated that the proposed sensor exhibits a high sensitivity, which is related to a CSRR having been used as the sensing element (as discussed at the beginning of Section 2). Nevertheless, note that the value that should be compared is the one that is expressed as a percentage, i.e., *S_av,f_*, where the relative variation in frequency is considered. The reason is that at high frequencies, the absolute variation of the notch frequency with the dielectric constant is larger. It is remarkable that *S_av,f_* is larger in the sensors of references [15,36] and in this work. The reason is that the average sensitivity is taken in a different range of dielectric constants. That is, in references [10,11,12,23,37,38], mixtures of DI water and ethanol are considered, with variations in dielectric constant between roughly 30 and 80. This high dielectric constant span explains the lower values of the sensitivity compared to those in [15,36] and those in this work, where oil samples with much smaller dielectric constants are considered. Consequently, a true and reliable comparison can only be made if the samples under test are comparable. In this regard, it can be seen that the sensor presented by Kulkarni et al. in [36] exhibits the highest value of *S_av,f_*, but at the expense of a more complex sensing device (that uses two ring resonators at different metal levels). By contrast, the sensor proposed in this work is simple; it uses a single metal layer (excluding the ground plane), its size is small, and it exhibits a good sensitivity, with *S_av,f_* = 3.58%. This value is larger than the one reported by Galindo-Romera et al. in [15], where the sensing device is based on a pair of split ring resonators (SRRs) loading a microstrip line etched in a single metallic layer.

One issue that is not present in our case, since we are not considering the characterization of solid slabs, is the air gap effect [4,5,6]. This effect appears as long as an air gap between the sample and the sensing element (resonator) occurs, which is inevitable when a solid sample is considered and put in contact (as required) with the sensing resonator (CSRR in our case). However, with liquids, two main problems may arise: (i) the presence of bubbles; and (ii) absorption by the substrate. The first problem can be minimized by properly treating/processing the liquid (e.g., by using vacuum chambers). In general, absorption can be minimized by using low-absorption materials and/or by protecting the sensing element with a dry film [23]. The latter approach is very effective, but in our sensing method it cannot be applied, since we need a homogeneous material at both sides of the CSRR slots. Liquid absorption by the substrate depends on the viscosity of the LUT; moreover, absorption does not proceed instantaneously. In relatively diluted liquids, such as DI water or ethanol, absorption arises, and measurements must be done immediately after introducing the LUT in the container. Such a procedure was done by Su et al. in [14], where the characterization of DI water was carried out. Absorption with DI water and the considered substrate was a relatively slow process. However, it was not possible to perform reliable measurements with ethanol. In oil samples, the viscosity is high, and absorption is very slow. Therefore, the proposed analytical method is specially suited for the characterization of oils and other viscous liquids.

## 5. Conclusions

In conclusion, we have presented and discussed in detail a sensing method for the estimation of the complex dielectric constant of liquids, which is based on a CSRR-loaded embedded microstrip line. The method is based on an analytical approach, and does not require calibration. Material parameters are simply inferred from the measured frequency response of the structure loaded with the liquid under test (LUT). We have applied this method for the determination of the dielectric constant and loss tangent of oils, and the results obtained are in reasonable agreement with the results inferred from independent measurements using dielectric characterization tools. Therefore, the method has been experimentally validated. The proposed method is fast and valid for the estimation of the complex permittivity of liquids, avoiding calibration. A more accurate determination of the complex permittivity would require alternative methods based on calibration curves, similar to those reported in the recent literature.

## Figures and Tables

**Table 1:** Circuit parameters corresponding to the empty sensing structure of Figure 1.

*L* (nH)	*C* (pF)	*C_c_* (pF)	*L_c_* (nH)	*C_c_subs_* (pF)	*R_s_* (Ω)	*R_D_* (Ω)	R_M_ (Ω)
3.70	1.09	8.07	0.43	7.35	25,015	3709	0.0147

**Table 2:** Comparison of various sensors in terms of sensitivity.

Reference	*f*_0_ (GHz)	*S_av_* (MHz)	*S_av,f_* (%)	Measured Permittivity Range (*ε*′)/(*εʺ*)
[15]	1.8	–	3.04	2.45–22.52/0.0387–0.939
[10]	2	4.76	0.238	9–79.5/9–10
[11]	1.9	1.53	0.081	9–80/10–13
[12]	3.5	9.16	0.261	6.5–80/0.1–0.4
[23]	0.87	0.79	0.091	27.86–80.86/3.04–10
[36]	2.5	8	0.32	2–76.7/0.06–10.27
[37]	20	59.75	0.298	20–44.7/23.7–33.8
[38]	2.2	–	5.89	1.88–2.12/0.0094–0.016
This work	2.5	89.5	3.58	2.93–3.64/0.3–0.5
